# Pharmacological basis for the use of *Hypericum oblongifolium* as a medicinal plant in the management of pain, inflammation and pyrexia

**DOI:** 10.1186/s12906-016-1018-z

**Published:** 2016-02-01

**Authors:** Naila Raziq, Muhammad Saeed, Muhammad Shahid, Naveed Muhammad, Haroon Khan, Farah Gul

**Affiliations:** 1 Department of Pharmacy, University of Peshawar, Peshawar, 25120 Pakistan; 2 Department of Pharmacy, Sarhad University of Science and Information Technology, Peshawar, Pakistan; 3 Department of Pharmacy, Abdul Wali Khan University, Mardan, Pakistan

**Keywords:** *Hypericum oblongifolium*, Methanol extract, Antinociceptive, Anti-inflammatory, Antipyretic activities

## Abstract

**Background:**

The present therapeutic agents for the treatment of pain, inflammation and pyrexia are not very effective and accompanied by various side effects. Therefore, new effective agents are the most wanted. The present study investigates the anti-nociceptive, anti-inflammatory and antipyretic activities of crude methanol extract of *Hypericum oblongifolium*.

**Methods:**

In vivo acetic acid induced writhing and hot plate tests were used for antinociceptive effects at 100, 200 and 300 mg/kg i.p. The anti-inflammatory and antipyretic potential of methanol extract were tested in carrageenan induced paw edema in mice and yeast induced hyperthermia respectively.

**Results:**

The extract doses of 100, 200 and 300 mg/kg i.p. revealed significant inhibitory effect (*P* < 0.001) in acetic acid induced writhing test. Pretreatment of extract at doses of 100, 200 and 300 mg/kg i.p. produced significant anti-inflammatory effect (*P* < 0.001) in the carrageenan induced paw edema. The methanol extract also showed significant antipyretic effect in yeast induced hyperthermia in mice during various assessment times.

**Conclusions:**

The methanol extract of *H. oblongifolium* showed significant anti-nociceptive, anti-inflammatory and antipyretic effects in various animal models and thus validates the traditional uses of the plant in said conditions.

## Background

Pain, inflammation and pyrexia can result in unrelenting symptoms, misery, stress and sometimes disablement for the sufferer. Allopathic medicine uses drug treatments to decrease the inflammation and pyrexia as well as reduce the pain by blocking various inflammatory mediators especially the prostaglandins, leukotrienes, cytokines and histamine. However, these medications often cause side effects such as stomach bleeding, bone demineralisation, kidney damage and nutritional deficiencies [1, 2]. The use of complementary and alternative medicine has been shown to produce beneficial effect in the management of various painful inflammatory conditions [3, 4] and several natural remedies are available that are often as effective as drugs without the side effects [5, 6]. Thus there is an ample scope for new natural medicines to combat different pathological conditions associated with pain, inflammation and pyrexia.

*Hypericum*, is a large genus of the family Hypericacea and consists of herbs or shrubs that grow widely in the temperate regions. Useful drugs, dyes, pigments, timbers, gums and resins have been isolated from its different members [7]. This genus is widely studied for anti-depressant, anti-microbial, antioxidant, antiviral, anxiolytic, anticancer, anti-inflammatory and anti-ulcerogenic activities. Several species of this genus especially *Hypericum perforatum* [8], *Hypericum caprifoliatum* [9, 10], *Hypericum polyanthemum* [11, 12], and *Hypericum grandifolium* [13, 14] were extensively studied for their utility in different diseases like depression, seasonal effective disorder, HIV and hepatitis-C infection, obsessive compulsive disorder, as well as other pathological conditions associated with pain and inflammation. Their use in these conditions has been validated by using a number of different animal and in vitro models.

*Hypericum oblongifolium* Wall. (family: Hypericacea), is a 6–12 m high erect evergreen shrub that grows at an altitude of 5000-6000 ft and is common in China and Himalaya [15]. This plant has been traditionally used for the treatment of hepatitis, nasal hemorrhage, gastric ulcer, external wounds, sedative, antispasmodic, antiseptic and as a remedy for sting of bees and dog bites [16, 17]. *H. oblongifolium* has been tested for chymotrypsin [15] and urease inhibition [18], antioxidant, anti-glycation, anti-lipid peroxide inhibition [19], antispasmodic, bronchodilator, blood pressure lowering [16], anti-ulcer [20] and anti-proliferative activities [17]. Phytochemical analysis showed that *H. oblongifolium* contained flavonoids, saponins and tannins [16]. Recently, various chemical compounds have been isolated from this plant and include triterpenes like hyperinols A and B [15], flavonoids like quercetin, myricetin, rhamnetin, kaempferol, luteolin [21], 18-β-H-urs-20 (30)-en-3β-ol-28-oic acid, tetracosyl 3-(3,4-dihydroxyphenyl) acrylate, shikimic acid, 1-octatriacontanol, hexacosyl tetracosanoate, β-sitosterol and β-sitosterol 3-O-β-D-glucopyranoside [17], xanthones like hypericorin-C, hypericorin-D, 3,4-dihydroxy-5-methoxyxanthone, along with 2,3-dimethoxyxanthone, 3,4-dihydroxy-2-methoxyxanthone, 3,5-dihydroxy-1-methoxyxanthone, 3-acetylbetulinic acid, 10-*H*-1,3-dioxolo [4,5-*b*]-xanthen-10-one, 3-hydroxy-2-methoxyxanthone, 3,4,5-trihydroxyxanthone and betulinic acid [20]. More recently our research group reported a new antioxidant i.e. folicitin from *H. oblongifolium* [22].

Keeping in view the potential pain and inflammation relieving properties of the genus *Hypericum*, the present study has been attempted to explore the *Hypericum oblongifolium* methanol extract (HOME) for its prospective analgesic, anti-inflammatory and anti-pyretic activities.

## Methods

### Chemicals

Diclofenac sodium (Suzhou Ausun Chemical Co, Lit.,China), acetic acid, Tramadol (Searle Pakistan Ltd.), carrageenan (Sigma Lambda, USA), Brewer’s yeast (Merck Germany), paracetamol (Tianjin Bofa Pharmaceutical Co, Lit., China), commercial grade methanol was obtained from Haq chemicals Peshawar. Sterile normal saline was used in all experiments as control while the methanol extract was prepared in normal saline.

### Animals

BALB/c mice of either sex weighing 25–30 gm were purchased from the Pharmacology section of the Department of Pharmacy, University of Peshawar, Pakistan. The animals were maintained in a 12 h light/dark cycle at 22 ± 2 °C. Access to food and water was *ad libitum*. Experiments on animals were performed between 9:00 am and 3:00 pm. The experiment protocols were approved by the Ethical Committee of the Department of Pharmacy, University of Peshawar, Pakistan.

### Plant material

Whole plant of *Hypericum oblongifolium* was collected in April and May, 2011, from Bara Gali, Abbottabad, Khyber Pakhtunkhwa. The plant was identified by Prof. Dr. Muhammad Ibrar of the Department of Botany, University of Peshawar, Pakistan and a voucher specimen (08823) was deposited in the herbarium of the same department. The powdered plant material was extracted in 20 % methanol.

### Antinociceptive activity

#### Acetic acid induced writhing test

Animals were divided into five treatment groups with six animals per group. Group I was injected with normal saline (10 ml/kg) as control, Group II received diclofenac sodium (10 mg/kg) while groups III, IV and V were injected with 100, 200 and 300 mg/kg i.p. of HOME respectively. After 30 min of treatment with saline, diclofenac sodium and extract, the animals were injected with 1 % acetic acid i.p. The number of abdominal constrictions (writhes) were counted after 5 min of acetic acid injection for a period of 10 min [23].

#### Hot plat test

Animals were subjected to a pretest on hot plate (Havard apparatus) maintained at 55 ± 0.1 °C. During pre-testing, the animals having a latency time greater than 15 s were rejected. The mice were divided into five treatment groups with six animals per group. Group I was treated with saline (10 ml/kg), group II with Tramadol (30 mg/kg i.p) while groups III, IV and V were treated with 100, 200 and 300 mg/kg i.p HOME respectively. After 30 min of treatment, the animals were placed on the hot plate and the latency time (time for which mouse remains on the hot plate without licking or flicking of hind limb or jumping) was measured in sec after 30, 60 and 90 min. In order to prevent tissue damage, a cut-off time of 30 s was imposed for all the animals [23, 24]. The percent analgesia was calculated using the following formula:

$$ \%\ \mathrm{Analgesia} = \left(\mathrm{Test}\ \mathrm{latency}\ \hbox{--}\ \mathrm{Control}\ \mathrm{latency}\right)\ /\ \left(\mathrm{Cut}\ \mathrm{off}\ \mathrm{time}\ \hbox{--}\ \mathrm{Control}\ \mathrm{latency}\right) \times 100\Big] $$

### Anti-inflammatory activity

Animals were randomly divided into five treatment groups with six animals per group. Group I was treated with normal saline (10 ml/kg), group II with diclofenac sodium (10 mg/kg) and the rest of groups i.e. III, IV and V were treated with HOME at doses of 100, 200, and 300 mg/kg, i.p respectively. After 30 min of treatment, carrageenan (1 %, 0.05 ml) was injected subcutaneously in the sub plantar tissue of the right hind paw of each mouse. The inflammation was measured immediately after the first dose and then after 1, 2, 3 and 4 h of carrageenan using a digital plethysmometer (LE 7500 plan lab S.L). The average foot swelling readings from the drug and standard treated animals was compared with that of control [23, 24]. The percent inhibition was determined according to the formula.

$$ \mathrm{Percent}\ \mathrm{inhibition} = \mathrm{A}\ \hbox{--}\ \mathrm{B}/\mathrm{A} \times 100 $$

Where A is the paw volume of control and B is the paw volume of the tested group.

### Antipyretic test

Animals were divided into five treatment groups with six animals per group. All animals were fasted overnight but allowed free access to drinking water. The normal rectal temperature of each mouse was recorded using a digital thermometer and then pyrexia was induced in all mice by injecting 20 % aqueous suspension of Brewer’s yeast (10 ml/kg, s.c). After 24 h, rectal temperature of each mouse was recorded. The induction of pyrexia was confirmed by a rise in temperature of more than 0.5 °C and animals showing a rise in temperature less than 0.5 °C were excluded from the experiment [23, 25]. Group I received saline as a negative control, Group II received paracetamol (150 mg/kg) as a standard drug while the remaining groups III, IV and V received 100, 200 and 300 mg/kg i.p of HOME respectively. After drugs administration, rectal temperature was again recorded periodically at 1, 2, 3, and 4 h.

### Statistical analysis

Data were expressed as mean ± SEM. The statistical significance of the differences between groups was tested by one-way analysis of variance (ANOVA) followed by Dunnett’s *post hoc* test using GraphPad Prism 5 (GraphPad Software Inc. San Diego CA, USA).

## Results

### Antinociceptive effect

#### Effect of acetic acid induced test

**Fig. 1:**
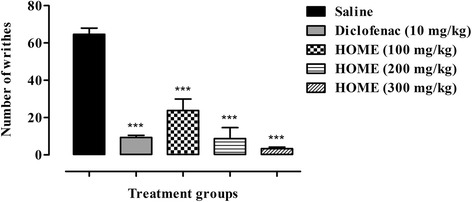
Antinociceptive activity of different doses of *Hypericum oblongifolium* methanol extract (HOME) (100, 200 and 300 mg/kg) in acetic acid induced writhing assay. The total number of writhes was expressed as mean ± SEM. ANOVA followed by Dunnett’s *post hoc* test. ***P < 0.001 compared to saline treated group, *n* = 6 mice/group

As shown in Fig. 1, HOME at all the tested doses (100, 200 and 300 mg/kg) significantly (*P* < 0.001) ameliorated the acetic acid induced writhes. The inhibition was comparable to that of standard, diclofenac sodium (*P* < 0.001). Maximum percent inhibition (95.98 %) of acetic acid induced pain was observed at 300 mg/kg dose of HOME.

#### Hot plate test

**Fig. 2:**
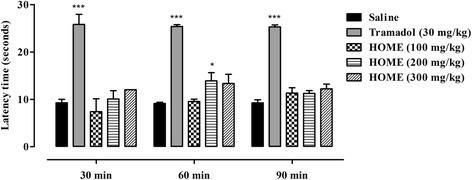
Antinociceptive activity of *Hypericum oblongifolium* methanol extract (HOME) (100, 200 and 300 mg/kg) in the hot plate test. Each bar represents mean ± SEM. ANOVA followed by Dunnett’s *post hoc* test. *P < 0.05, ***P < 0.001. *n* = 6 mice/group

As shown in Fig. 2, no significant effect was observed in the latency time by HOME except at a dose of 200 mg/kg (*P* < 0.05) after 60 min as compared to saline treated group. Significant increase (*P* < 0.001) in the latency time was noticed with the standard opioid analgesic (Tramadol) after 30, 60 and 90 min.

### Anti-inflammatory activity

**Fig. 3:**
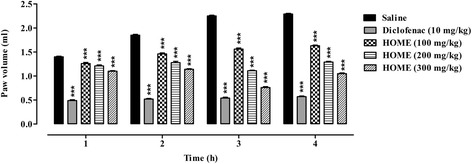
Anti-inflammatory activity of *Hypericum oblongifolium* methanol extract (HOME) (100, 200 and 300 mg/kg) in the carrageenan induced paw edema in mice. Each bar represents mean ± SEM. ANOVA followed by Dunnett’s *post hoc* test. ***P < 0.001. *n* = 6 mice/group

As shown in Fig. 3, significant protection (*P* < 0.001) from carrageenan induced inflammation was exhibited by HOME at doses of 100, 200 and 300 as compared to saline treated group. Likewise the standard, diclofenac sodium also produces significant reduction (*P* < 0.001) of carrageenan induced paw edema in the four hours testing paradigm.

### Antipyretic test

**Fig. 4:**
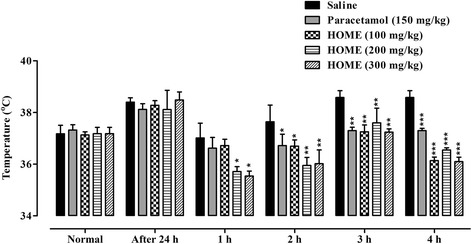
Antipyretic activity of *Hypericum oblongifolium* methanol extract (HOME) at 100, 200 and 300 mg/kg i.p. in yeast induced pyrexia. Each bar represents mean ± S.E.M. ANOVA followed by Dunnett’s *post hoc* test. *P < 0.05, **P < 0.01, ***P < 0.001 compared to saline treated group. *n* = 6 mice/group

As shown in Fig. 4, HOME provided significant protection against hyperthermia induced by yeast after 1 h of treatment at doses of 200 and 300 mg/kg (*P* < 0.05). Similarly, significant antipyretic effect was observed with all the tested doses i.e. 100, 200 and 300 mg/kg after 2 h (*P* < 0.05, *P* < 0.01), 3 h (*P* < 0.01) and 4 h (*P* < 0.001) of treatment. Likewise, the standard, paracetamol significantly decreased the febrile response after 2–3 h (*P* < 0.01) and 4 h (*P* < 0.001) of treatment.

## Discussion

The present study evaluated the analgesic, anti-inflammatory and antipyretic activities of the methanol extract of *H. oblongifolium*.

Acetic acid induced abdominal constriction assay is a well recommended protocol in evaluating medicinal agents for their peripheral analgesic activity [26, 27]. In this paradigm, pain is induced by the liberating endogenous substances especially the prostaglandins through the action of the constitutive enzyme cyclooxygenase-1 (COX-1) and its isoform COX-2. Induction of this mechanism through COX enzymes and stimulation of these sensory pathways in the mouse peritoneum incites a viscero-somatic reflex and the abdominal constrictions (writhing) [28]. The acetic acid induced writhing method is sensitive to analgesics and sensory afferents in the peritoneum carry α1/2-adrenoceptors, β-adrenoceptors and opioid receptors on their terminals. When activated by appropriate agonists, these receptors depress the generation of pain impulses [29, 30]. Regarding the results of our extract in acetic acid-induced abdominal constriction assay, a prominent inhibition of writhing reflex was observed. These findings strongly recommend that HOME has peripheral analgesic activity and their mechanisms of action might be mediated through inhibition of local peritoneal receptors or inhibition of cyclooxygenase enzymes. The writhing test however is non-specific in nature as it does not exactly indicate the involvement of central or peripheral mechanism. On the other hand, the hot plate method involves spinal reflex and may be considered as a suitable model for the determination of central anti-nociceptive mechanism [31–33]. Although, in this study, HOME produce significant analgesic effect in the hot plate test only at a dose of 200 mg/kg after 60 min (*P* < 0.05), however, a non-statistical significant protection against thermal induced nociception was observed at doses of 300 mg/kg (35.9 %) after 30 min, 100 mg/kg (13.9 %) and 300 mg/kg (34.9 %) after 60 min and for all the tested doses after 90 min i.e. 100 mg/kg (47.9 %), 200 mg/kg (71.8 %) and 300 mg/kg (51.6 %) (Fig. 2). In comparison the standard opioid analgesic i.e. tramadol produced significant analgesic effect (*P* < 0.001) after 30, 60 and 90 min.

Carrageenan-induced paw edema is a well established animal model to assess the anti-inflammatory effect of natural products as well as synthetic chemical compounds. Carrageenan-induced edema in paw is a biphasic event; the initial phase (1 or 1.5 h) is predominately a non-phagocytic edema followed by a second phase (2–5 h) with increased edema formation that remained up to 5 h. The initial phase has been attributed to the release of various mediators including histamine, serotonin and bradykinin while the late phase or second phase edema has been shown to be caused by overproduction of prostaglandins [34–36]. The result of pre-treatment of HOME demonstrated that the extract is effective in alleviating inflammation during the entire study duration. Significant protection against carragennan induced paw edema was afforded by the 100, 200 and 300 mg/kg doses of HOME after 1 h (10 %, 13.5 %, 21.4 %), 2 h (21 %, 30.8 %, 38.3 %), 3 h (30.6 %, 50.6 %, 66.2 %) and 4 h (28.8 %, 43.6 %, 54.1 %) of treatment. The ability to decrease paw inflammation in mice by all the tested doses of HOME was significantly (*P* < 0.001) comparable to that of the standard, diclofenac sodium (10 mg/kg) (Fig. 3).

Subcutaneous injection of Brewer’s yeast induces pyrexia by increasing the synthesis of prostaglandins and is considered as a useful test for screening of plants materials as well as synthetic drugs for their antipyretic effect [37, 38]. The inhibition of prostaglandin synthesis among other mediators can be regarded as a possible mechanism of antipyretic action like that of paracetamol which inhibits the synthesis of prostaglandin by blocking the COX enzyme activity [39, 40]. In this study, the intraperitoneal administration of HOME significantly attenuated the hyperpyrexia induced by yeast in mice. All the doses of HOME (100, 200 and 300 mg/kg) were able to reduced the febrile response and the protection is comparable to the standard paracetamol especially after 2 h and remained effective throughout the study duration (4 h).

Studies showed that *H. oblongifolium* contains xanthones which possess strong *in vitro* anti-inflammatory [41] and anti-ulcer [20] activities by inhibiting the respiratory burst of neutrophils and urease respectively. Luteolin and myricetin, which are the most abundant flavonoid aglycones along with quercetin, rhamnetin, and kaempferol present in *H. oblongifolium* [21] are known to have analgesic [42], anti-inflammatory [43] and antipyretic [44] activities. Moreover, strong antioxidants including folicitin [22] and quercetin have been isolated from *H. oblongifolium* and antioxidants are able to reduce pain, pyrexia and inflammation in both animal models [45–47] as well as in humans [48, 49]. Furthermore, other *Hypericum* species including the widely used official medicine, *Hypericum perforatum* are reported to have strong antidepressant, antinociceptive, antiviral, antimicrobial, antipyretic, anti-inflammatory and healing properties [50].

## Conclusions

*H. oblongifolium* methanol extract was evaluated for its anti-nociceptive, anti-inflammatory, and antipyretic potential. The crude extract possessed significant peripheral analgesic activity as well as attenuated the carrageenan induced inflammation and yeast induced pyrexia.
